# Irritable bowel syndrome-like symptoms and health related quality of life two years after Roux-en-Y gastric bypass - a prospective cohort study

**DOI:** 10.1186/s12876-019-1103-0

**Published:** 2019-12-02

**Authors:** Ingvild Kristine Blom-Høgestøl, Martin Aasbrenn, Monica Chahal-Kummen, Cathrine Brunborg, Inger Eribe, Jon Kristinsson, Per G. Farup, Tom Mala

**Affiliations:** 1 Department of Endocrinology, Morbid Obesity and Preventive Medicine, Oslo University Hospital, Oslo, Norway; 2 Institute of Clinical Medicine, University of Oslo, Oslo, Norway; 3 Department of Geriatrics and Internal Medicine, Bispebjerg and Frederiksberg Hospital, Copenhagen, Denmark; 4 Department of Surgery, Innlandet Hospital Trust, Gjøvik, Norway; 5 Oslo Centre for Biostatistics and Epidemiology, Research Support Services, Oslo University Hospital, Oslo, Norway; 6 Department of Gastrointestinal Surgery and Paediatric Surgery, Oslo University Hospital, Oslo, Norway; 7 Unit for Applied Clinical Research, Department of Clinical and Molecular Medicine, Faculty of Medicine and Health Sciences, Norwegian University of Science and Technology, Trondheim, Norway; 8 Department of Research, Innlandet Hospital Trust, Brumunddal, Norway

**Keywords:** Irritable bowel syndrome, Morbid obesity, Roux-en-Y gastric bypass, Gastrointestinal symptoms, Fibromyalgia, Health related quality of life

## Abstract

**Background:**

Irritable bowel syndrome (IBS) is prevalent in patients with morbid obesity. After Roux-en-Y gastric bypass (RYGB) chronic abdominal pain is common, however the etiology is largely unknown. We aimed to study the change in the prevalence of IBS-like symptoms 2 years after RYGB and possible preoperative predictors of such symptoms. Secondly, to evaluate changes in symptoms of constipation and diarrhea, and Health related quality of life (HRQoL).

**Methods:**

Patients with morbid obesity were included at two obesity centers in South-Eastern Norway. IBS was diagnosed according to the Rome III criteria. Predictors were evaluated in a multivariable logistic regression analysis.

**Results:**

Of 307 participants operated with RYGB, 233 (76%) completed the study questionnaires. Preoperatively 27/233 participants (12%) had IBS, 2 years after RYGB 61/233 (26%) had IBS-like symptoms (*p* < 0.001). Eleven participants with IBS preoperatively (41%) did not report such symptoms after RYGB. New onset IBS-like symptoms was identified in 45/206 (22%) after RYGB. Fibromyalgia, low LDL levels, high vitamin B_1_ levels and IBS before RYGB were independent preoperative predictors of IBS-like symptoms at the follow-up visit. Symptom scores for constipation preoperatively and 2 year after RYGB were 1.5 (0.9) and 1.8 (1.2), and for diarrhea 1.4 (0.9) and 1.8 (1.1), respectively (*p* < 0.001). We observed a significant improvement in the physical component score for all participants. However, participants with new onset IBS-like symptoms had a significant worsening of the mental component score.

**Conclusions:**

The prevalence of IBS-like symptoms doubled 2 years after RYGB, and these symptoms were associated with reduced HRQoL. Preoperative IBS and fibromyalgia were strong predictors of postoperative IBS-like symptoms.

## Background

Irritable bowel syndrome (IBS) affects around 10% of adult populations [1]. IBS might be even more common in patients with morbid obesity [2, 3]. The core symptom of IBS is frequent abdominal pain associated with changes in bowel habits. The underlying pathophysiology is multifactorial and includes a combination of psychological factors and gut related factors (as changes in motility, inflammation or the gut microbiome) [4]. The diagnosis IBS is based on symptom-based criteria [5].

Roux-en-Y gastric bypass (RYGB) is the second most common surgical procedure applied in the treatment of morbid obesity when conservative measures have failed [6]. RYGB enables a large and sustained weight loss, improvement of obesity-related co-morbidities and health related quality of life (HRQoL), with low risk of surgical complications [7–9]. However, recent reports indicate that abdominal pain is common 2–5 years after RYGB [10–12]. Internal herniation and gallstone related disease are common causes of acute pain, whereas the etiology of chronic abdominal pain after RYGB is less well understood [13–15].

The primary aims of this study were to study the change in the prevalence of IBS-like symptoms from before to 2 years after RYGB and to search for preoperative predictors of these symptoms after RYGB. Furthermore we aimed to study changes in scores of constipation, diarrhea and HRQoL in all participants and in those with and without IBS-like symptoms.

## Methods

### Study design and setting

This is a prospective cohort study, where we invited patients with morbid obesity at the obesity centers at Oslo University Hospital (OUH) and Innlandet Hospital Trust Gjøvik (IHT-G), Norway, to participate. Participants who underwent RYGB were invited for a follow-up consultation 2 years after RYGB. Participants at OUH and IHT-G were recruited during February 2014 to June 2015, and December 2012 to September 2014, respectively. Follow-up was completed June 2017 and May 2017 at OUS and IHT-G, respectively. Subject with morbid obesity who previously has failed non-surgical measures of weigh loss were considered eligible for. Morbid obesity was defined as having a body mass index (BMI) ≥ 40 kg/m^2^ or BMI ≥ 35 kg/m^2^ with obesity-related comorbidity. Exclusion criteria for study inclusion were age younger than 18 or older than 60 years, previous bariatric surgery, other indications for RYGB than morbid obesity, simultaneous cholecystectomy, other serious disorders unrelated to obesity (including organic gastrointestinal disorder) and inability to read Norwegian language.

### Surgical procedure and study visits

All RYGB operations were performed in a standardized manner, with a laparoscopic approach, a gastric pouch of about 25 ml, a 150 cm antecolic alimentary and a 50 cm biliopancreatic limb [16]. Mesenteric defects were routinely closed with non-absorbable staplers. Study visits were performed preoperatively and 2 years after RYGB. At study visits, demographics, medical history, and were registered in a predefined case report form. A routine clinical examination was performed including anthropometric measurements, and fasting blood samples were retrieved. If symptoms or clinical examination was susceptive of organic gastrointestinal disorder additional diagnostic evaluations including endoscopic examinations were made according to the clinician’s discretion. All patients filled in questionnaires for the classification of IBS, gastrointestinal symptoms and HRQoL.

### Outcomes

Five demographic variables were evaluated: Age (years), gender (male/female), BMI (kg/m^2^), employment status (full time/not working) and cohabitant status (living with partner/not living with partner). Five co-morbid conditions were evaluated: Type 2 diabetes (T2D), hypothyroidism, hypertension, fibromyalgia, and minor psychiatric disorders (anxiety or depression). The information concerning co-morbidity was given by the participant and reviewed by a clinician with full access to the patient’s medical record. T2D was defined as HbA_1c_ ≥ 6.5%, or the use of one or more oral glucose lowering drugs, with or without insulin treatment. Diabetes remission was defined as HbA_1c_ < 6.5% without use of glucose lowering drugs, in a participant with prior T2D.

### IBS, IBS-like symptoms, gastrointestinal symptoms and HRQoL

IBS and IBS-like symptoms were evaluated by the Rome III questionnaire in a validated translation to Norwegian [5]. Preoperatively participants who fulfilled the Rome III criteria for IBS were classified as having IBS, postoperatively participants who fulfilled the Rome III criteria for IBS were classified as having IBS-like symptoms. Our study protocol did not include supplementary examinations of participants fulfilling the Rome III criteria to explore underlying pathophysiology of IBS or IBS-like symptoms. Rome III evaluates pain and discomfort without differentiating these measures. Furthermore, participants reporting preoperative IBS did not receive any treatment addressing IBS symptoms as part of this study or to our knowledge by others. Thus the presented IBS population likely represents an untreated IBS population. Notably, no restrictions were placed on participants in regard to seeking medical attention for IBS related symptoms.

Symptoms of constipation and diarrhea were evaluated using the Gastrointestinal Symptoms Rating Scale (GSRS). The patients responded using a Likert-type scale (1 = no discomfort and 7 = severe discomfort) [17]. The GSRS-IBS questionnaire was used at IHT-G, and the GSRS at OUS. Common variables from the two questionnaires were used to calculate the constipation and diarrhea symptom scores reported in this article.

Participants from OUS reported HRQoL at the preoperative and 2 year follow-up visit, participants from IHT-G reported HRQoL only at the 2 year follow-up visit. To evaluate HRQoL the Norwegian validated translation of the 36-Item Short Form Health Survey (SF-36) version 2 was used [18]. The SF-36 questionnaires were scored using the QualityMetric Health Outcomes™ Scoring Software 4.5 giving eight health domains; physical functioning, physical role functioning, bodily pain, general health perception, vitality, social role functioning, emotional role functioning and mental health, and two summary scores; physical component score and mental component score.

### Blood tests

The reference values for the fifteen variables reported in the results are as follows: *hemoglobin* g/dL: women 11.7–15.3, men 13.4–17.0; *iron* μmol/L: 9–34; *ferritin* μg/L: women 10–170, men 30–400; *white-cell count* 10^9^/L: 3.5–10.0; *c-reactive protein (CRP)* mg/L: < 5; *cholesterol* mmol/L: age 18–29 2.9–6.1, age 30–49 3.3–6.9, age > 50 3.9–7.8; *high-density lipoprotein (HDL)* mmol/L: women 1.0–2.7, men 0.8–2.1; *low-density lipoprotein (LDL)* mmol/L: age 18–29 1.3–4.3, age 30–49 1.5–4.8, age > 50 2.0–5.4; *triglycerides* mmol/L: 0.5–2.6; *thyroid stimulating hormone (TSH)* mIE/L: 0.5–3.6; *vitamin B*_*1*_ nmol/liter: 95–200; *vitamin B*_*6*_ nmol/liter: 15–160; *vitamin B*_*12*_ pmol/L: 150–650; *folic acid* nmol/L: 7–40; glycosylated *hemoglobin* (*HbA*_*1c*_) %: 4.0–6.0.

### Statistical analysis

Paired-sample t–tests or Wilcoxon rank test were used for evaluation of changes in continuous variables between preoperative and 2 year follow-up. McNemar’s test for paired proportions was used for evaluation of changes in categorical variables. For comparing subgroups independent sample t-test was used. Missing data for the GSRS item scores was imputed using the mean score of the non-missing items. Patient characteristics associated with IBS-like symptoms were studied by using independent sample t-test, the Chi Square test or Fisher exact test as appropriate.

Logistic regression analysis was performed to identify preoperative predictors of IBS-like symptoms 2 years after RYGB. Any variable associated with *p* < 0.25 from the univariable analysis were entered into a multivariable logistic regression model using a manual backward stepwise elimination procedure. Centre of inclusion and IBS at baseline were forced into the multivariable model. Multivariable analyses were preceded by estimation of correlation between risk factors. Predictors that correlated > 0.7, were not included in the model in order to avoid multicollinearity. The association between preoperative characteristics and IBS-like symptoms at the 2 year follow-up visit was quantified by odds ratio (OR) with 95% confidence intervals (CI). Two tailed *p*-values < 0.05 were considered statistically significant. All statistical analyses were made using the IBM SPSS statistics version 25.0 (IBM SPSS Inc., Armonk, NY: IBM Corp). The results are given as mean with standard deviation (SD) in brackets if not otherwise indicated.

## Results

**Fig. 1:**
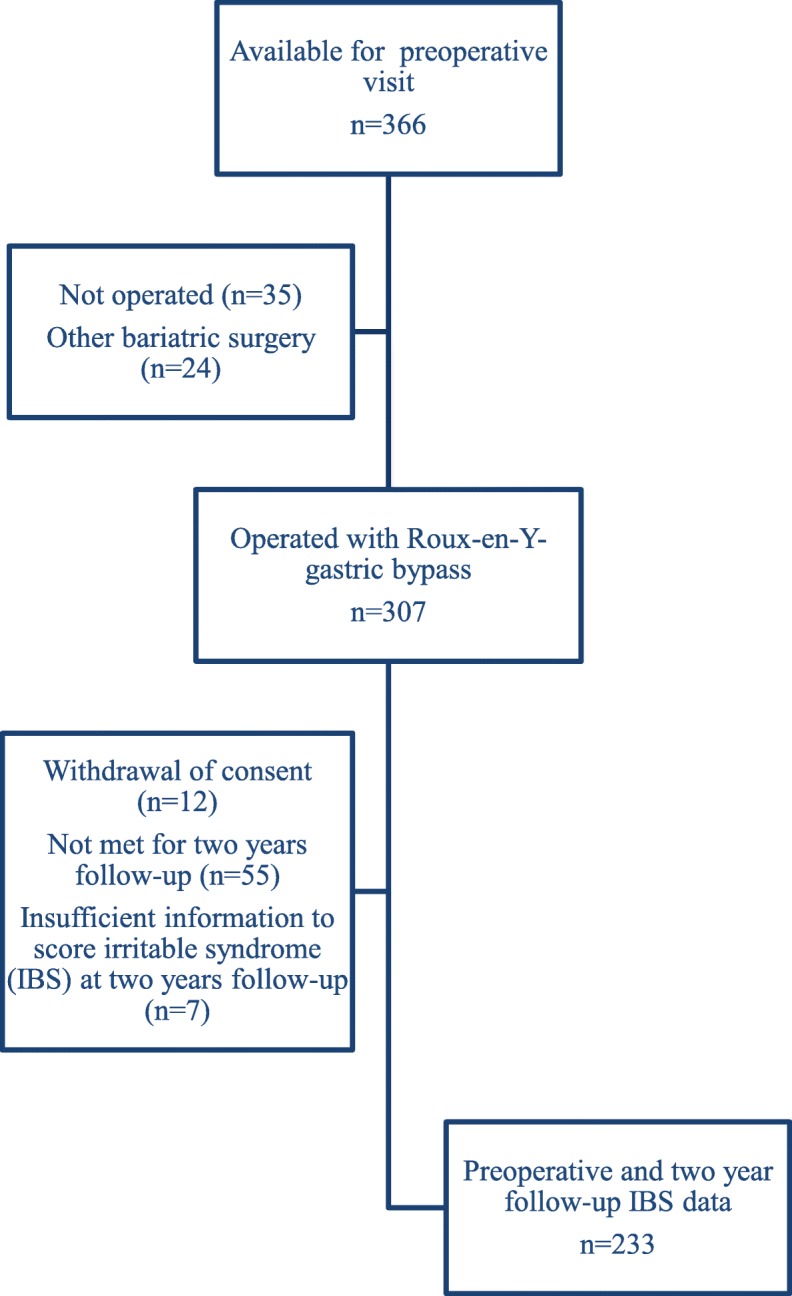
Flow chart describing the inclusion of participants

Of 307 participants operated with RYGB, 240 (78%) attended the 2 year follow-up visit and 233 (76%) filled out the study questionnaires (Fig. 1). In total 45 (19%) of 233 included participants were operated at IHT-G. Table 1 gives the participants’ characteristics at the preoperative and the 2 year follow-up visits.

**Table 1:** Patient characteristics at the preoperative visit and the 2 year follow-up visit after Roux-en-Y-gastric bypass

	Preoperative visit *n* = 233	Two year follow-up visit *n* = 233	Statistics (*p*-values)
Gender (male/female)	56 (24%)/177 (76%)		
Age (years)	43.5 (9.1)		
Body mass index (kg/m^2^)	42.9 (4.9)	29.5 (4.6)	**<0.001**
Living with partner	161 (71%)	172 (76%)	**0.03**
Full time work	130 (58%)	127 (56%)	0.78
Type 2 diabetes	55 (24%)	18 (8%)	**<0.001**
Hypertension	98 (43%)	34 (15%)	**<0.001**
Hypothyroidism	22 (10%)	24 (10%)	0.73
Fibromyalgia	33 (14%)	36 (16%)	0.61
Self-reported psychiatric disorder	40 (17%)	37 (16%)	0.76
Irritable bowel syndrome	27 (12%)	61 (26%)	**<0.001**
IBS-constipation	5 (2%)	11 (5%)	0.15
IBS-diarrhea	5 (2%)	12 (5%)	0.09
IBS-mixed	16 (7%)	34 (15%)	**0.004**
IBS-unsubtyped	1 (0.4%)	3 (1%)	0.63
Hemoglobin (g/dl)	14.2 (1.1)	13.7 (1.1)	**<0.001**
Iron (mol/L)	15.1 (5.2)	19.6 (6.9)	**<0.001**
Ferritin	104.5 (8–584)	115.0 (4–804)	0.31
White-cell count (×10^9^/liter)	7.7 (2.1)	6.2 (1.9)	**<0.001**
HbA_1c_ (%)	5.7 (4.6–14.9)	5.2 (4.0–9.7)	**<0.001**
C-reactive protein (mg/liter)	7.0 (0–50.0)	0.6 (0–66.0)	**<0.001**
Cholesterol (mmol/liter)	4.9 (1.0)	4.3 (0.7)	**<0.001**
High-density lipoprotein (mmol/liter)	1.2 (0.3)	1.7 (0.4)	**<0.001**
Low-density lipoprotein (mmol/liter)	3.2 (0.9)	2.5 (0.7)	**<0.001**
Triglycerides	1.9 (1.3)	1.0 (0.5)	**<0.001**
Vitamin B_1_ (nmol/liter)	157.4 (27.8)	159.6 (27.7)	0.18
Vitamin B_6_ (nmol/liter)	36.5 (34.9)	52.8 (44.0)	**<0.001**
Vitamin B_12_ (pmol/liter)	375.8 (166.8)	531.4 (321.3)	**<0.001**
Folic acid (nmol/liter)	20.3 (9.0)	22.2 (10.6)	**0.014**

The results are given as number (proportion in per cent) for categorical variables, mean (SD) for continuous variables with normal distribution and median (min-max) for other variables. Significant *p*-values are marked in bold

Data were analyzed with McNemar test for categorical variables and paired t-tests or Wilcoxon signed-rank test (marked with†) for continuous variables

For one patient, we had insufficient information for subtyping of IBS at the 2 year follow-up visit

Missing variables: Living with partner (*n* = 7), Full time work (*n* = 7), type 2 diabetes (*n* = 3), Hypertension (*n* = 4) Hypothyroidism (*n* = 4), Fibromyalgia (*n* = 4), minor psychiatric disorder (*n* = 4), Hemoglobin (=2), Iron (*n* = 2), Ferritin (*n* = 7), White-cell count (*n* = 3), HbA1c (n = 2), CRP (*n* = 3), Cholesterol (*n* = 2), High-density lipoprotein (*n* = 2), Low-density lipoprotein (n = 2), Triglycerides (*n* = 2), Vitamin B1 (*n* = 4), Vitamin B6 (*n* = 4), Vitamin B12 (*n* = 2), Folic acid (*n* = 2)

*Two de-novo diabetes mellitus

**Fig. 2:**
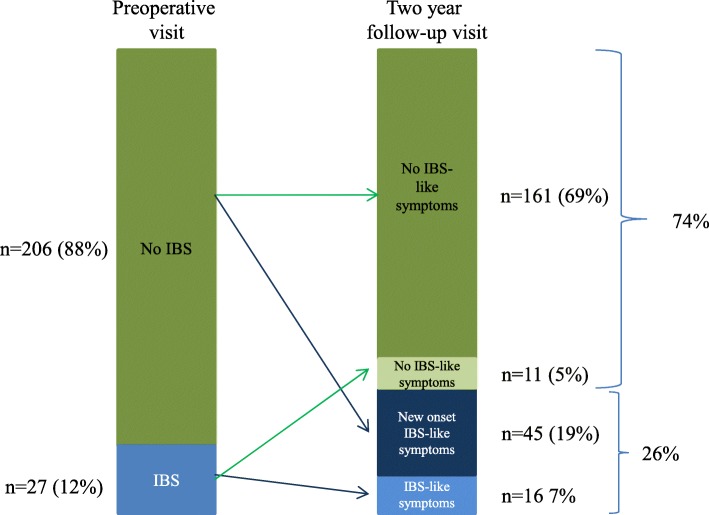
Prevalence of Irritable bowel syndrome (IBS) at the preoperative visit and IBS-like symptoms at the 2 year follow-up visit after Roux-en-Y-gastric bypass. IBS status was defined according to the Rome III criteria. The prevalence of symptoms of IBS increased from 12 to 26% (*p* < 0.001)

Prior to the RYGB surgery 27 participants (12%) had IBS, 2 years after surgery 61 (26%) reported IBS-like symptoms (*p* < 0.001). 11/27 (41%) of those with IBS before RYGB did not report IBS-like symptoms after RYGB. New onset IBS-like symptoms were identified in 45/206 (22%) of participants without IBS preoperatively (Fig. 2), representing 19% of the total population at the 2 year follow-up visit.

Fibromyalgia, low LDL levels, high vitamin B_1_ levels and IBS before RYGB were independent preoperative predictors of IBS-like symptoms at the 2 year follow-up visit (Table 2). Among participants without IBS at the preoperative visit, fibromyalgia and high vitamin B1 levels were independent preoperative predictors of IBS-like symptoms at follow-up (data not shown).

**Table 2:** Preoperative predictors of irritable bowel syndrome (IBS)-like symptoms 2 years after Roux-en-Y-gastric bypass (RYGB)

Preoperative characteristics	Patients without IBS-like symptoms after RYGB *n* = 172	Patients with IBS-like symptoms after RYGB *n* = 61	Odds ratio (95% CI)	Statistics (*p*-values)	Adjusted for all significant predictors and center of inclusion	
					Odds ratio (95% CI)	Statistics (*p*-values)
*Gender (female)*	*127/172 (74%)*	*50/61 (82%)*	*1.6 (0.77–3.4)*	***0.20***		
Age (years)	43.3 (9.3)	43.3 (8.4)	1.0 (0.94–1.0)	0.66		
Body mass index (kg/m^2^)	42.8 (4.9)	43.1 (4.6)	1.0 (0.96–1.1)	0.65		
*Living with partner*	*118/171 (69%)*	*47/61 (77%)*	*1.5 (0.77–3.0)*	***0.24***		
*Working full time*	*105/171 (61%)*	*28/61 (46%)*	*0.53 (0.30–0.96)*	***0.037***		
Smokers	39/168 (23%)	10/59 (17%)	0.68 (0.31–1.5)	0.32		
Type 2 diabetes	42/172 (24%)	15/61 (25%)	1.0 (0.51–2.0)	0.98		
Hypertension	74/171 (43%)	25/61 (40%)	0.76 (0.50–1.7)	0.76		
Hypothyroidism	14/172 (8%)	8/61 (13%)	1.7 (0.68–4.3)	0.26		
***Fibromyalgia***	***16/171 (9%)***	***17/61 (28%)***	***3.7 (1.8–8.0)***	***0.001***	***4.1 (1.7–9.8)***	***0.001***
*Self-reported psychiatric disorder*	*26/172 (15%)*	*14/61 (23%)*	*1.7 (0.81–3.5)*	***0.17***		
***IBS before RYGB***	***11/172 (6%)***	***16/61 (26%)***	***5.2 (2.6–12.0)***	***< 0.001***	***7.2 (2.8–18.6)***	***< 0.001***
Hemoglobin (g/dl)	14.1 (1.1)	14.3 (1.1)	1.1 (0.86–1.5)	0.41		
Iron (μmol/L)	15.0 (5.3)	15.6 (5.1)	1.0 (0.97–1.1)	0.43		
Ferritin (μg/L)	144 (130.3)	138 (103.8)	1.0 (0.99–1.0)	0.74		
White-cell count (×10^9^/liter)	7.6 (2.1)	7.7 (2.1)	1.0 (0.88–1.8)	0.83		
HbA_1_C (%)	6.1 (1.4)	6.0 (1.4)	0.94 (0.75–1.8)	0.58		
*Thyroid stimulating hormone (x10E-3 IU/L)*	*1.7 (0.95)*	*2.2 (1.9)*	*1.3 (1.0–1.7)*	***0.039***		
C-reactive protein (mg/liter)	7.7 (6.3)	7.7 (5.5)	1.0 (0.95–1.1)	0.99		
Cholesterol (mmol/liter)	5.0 (1.0)	4.8 (0.95)	0.82 (0.61–1.1)	0.20*		
High-density lipoprotein (mmol/liter)	1.2 (0.31)	1.2 (0.25)	0.86 (0.32–2.4)	0.77		
***Low-density lipoprotein (mmol/liter)***	***3.2 (0.92)***	***3.0 (0.75)***	***0.81 (0.58–1.1)***	***0.20***	***0.66 (0.45–0.96)***	***0.031***
Triglycerides (mmol/L)	1.86 (1.4)	1.88 (1.2)	1.0 (0.81–1.3)	0.93		
***Vitamin B***_***1***_ ***(nmol/liter)***	***155 (28.1)***	***162 (27.2)***	***1.0 (0.99–1.0)***	***0.11***	***1.0 (1.0–1.0)***	***0.013***
Vitamin B_6_ (nmol/liter)	36.6 (36.9)	36.0 (27.9)	1.0 (0.99–1.0)	0.91		
*Vitamin B*_*12*_ *(pmol/liter)*	*384 (182)*	*350 (110)*	*1.0 (1.0–1.0)*	***0.17***		
Folic acid (nmol/liter)	20.0 (9.0)	20.9 (8.8)	1.0 (0.98–1.0)	0.50		

The results are given as number (proportion in per cent) for categorical variables, and mean (SD) for continuous variables with normal distribution. Preoperative characteristics associated with IBS-like symptoms 2 years after RYGB were studied with univariate regression. Any variable associated from the univariate analysis (with p < 0.25) were entered into a multivariable logistic regression model using a manual backward stepwise elimination procedure. In total, 10 potential predictors were examined in the multivariable analysis (*p*-values written in bold). Cholesterol was not included in the final analysis due to high correlation with LDL

Symptom scores for constipation at the preoperative and 2 year follow-up visits were 1.5 (0.9) and 1.8 (1.2) (*p* < 0.001), and for diarrhea 1.4 (0.9) and 1.8 (1.1) (*p* < 0.001), respectively. Table 3 shows the changes in constipation and diarrhea scores in all participants, and stratified by IBS status. The most pronounced worsening of constipation and diarrhea symptom scores was found in the groups without IBS before RYGB and the group with new onset IBS-like symptoms after RYGB.

**Table 3:** Changes in constipation and diarrhea symptom scores from preoperatively to 2 years after Roux-en-Y Gastric bypass (RYGB)

Group	n	Δ constipation symptom score	95% CI	*p*-value	n	Δ diarrhea symptom score	95% CI	*p*-value
All participants	174	−0.29	−0.44 to −0.14	**<0.001**	177	−0.40	−0.58 to −0.22	**<0.001**
Subgroups based on IBS status preoperatively:								
With IBS	21	−0.19	−0.66 to 0.28	0.41	21	−0.38	−1.1 to 0.34	0.28
Without IBS	153	−0.31	−0.46 to −0.15	**<0.001**	156	−0.40	−0.58 to −0.22	**<0.001**
Subgroups based on IBS like symptoms 2 years after RYGB:								
Never IBS or IBS-like symptoms	116	−0.16	−0.32 to 0.012	0.068	119	−0.31	−0.50 to − 0.11	**0.002**
New onset IBS-like symptoms	37	−0.77	−1.13 to − 0.42	**<0.001**	37	− 0.71	− 1.15 to − 0.27	**0.002**
IBS preoperatively and IBS-like symptoms at 2 year follow-up visit	14	− 0.24	− 0.75 to 0.28	0.34	14	− 0.24	− 1.3 to 0.78	0.62

All participants, and subgroups based on status of irritable bowel syndrome (IBS) are presented. Significant *p*-values are marked in bold

The presented delta values: Gastrointestinal Symptom Rating Scale (GSRS) at the 2 year follow-up visit - GSRS at the preoperative visit. Positive delta values indicate improved symptoms after RYGB, negative delta values indicate worsened symptoms after RYGB. The results are given as mean values with 95% confidence intervals (CI). Data were analyzed with paired t-tests

New onset IBS: no IBS preoperatively, but IBS-like symptoms 2 years after RYGB. Never IBS: No IBS preoperatively or IBS-like symptoms 2 years after RYGB

Missing data Δ constipation symptom score: All participants; *n* = 59. Subgroups based on IBS status preoperatively: with IBS; *n* = 6, without IBS; *n* = 53. Subgroups based on IBS-like symptoms 2 years after RYGB: never IBS or IBS-like symptoms; *n* = 45, new onset IBS-like symptoms; *n* = 8, IBS at preoperative and IBS-like symptoms 2 year follow-up visit; *n* = 2

Missing data Δ diarrhea symptom score: All participants; *n* = 56. Subgroups based on IBS status preoperatively: with IBS; *n* = 6, without IBS; *n* = 50, never IBS; *n* = 42. Subgroups based on IBS-like symptoms 2 years after RYGB: new onset IBS-like symptoms; *n* = 8, IBS at preoperative and IBS-like symptoms 2 year follow-up visit; *n* = 2

**Fig. 3:**
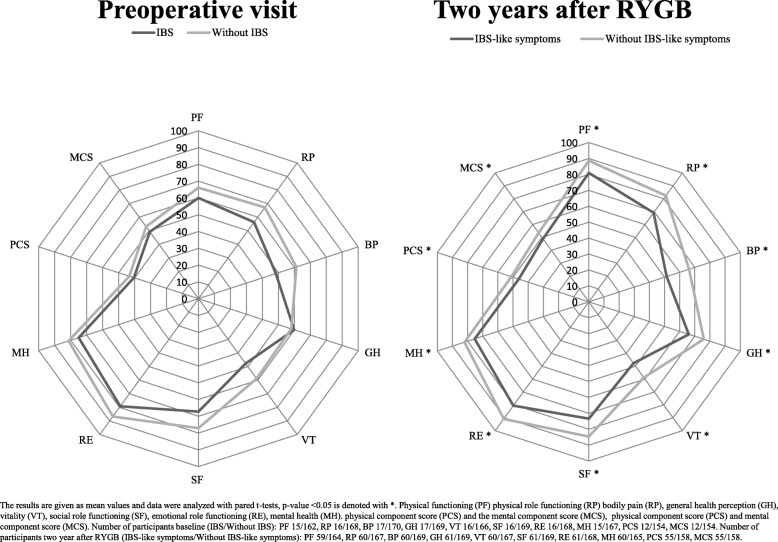
Health related quality of life in participants with and without irritable bowel syndrome (IBS) and IBS-like symptoms, before and 2 years after Roux-en-Y gastric bypass (RYGB)

Prior to RYGB, HRQoL was comparable between participants with and without IBS. At the 2 year follow-up visit participants with IBS-like symptoms had significantly lower HRQoL scores in all eight health domains and both summary scores compared to participants without IBS-like symptoms (Fig. 3). In participants with and without IBS at the preoperative visit, the improvements in the mean physical component score were 0.54 (12.8) and 7.6 (10.6) (*p* = 0.047) respectively and mental component scores decreased with 1.2 (10.7) and 2.6 (8.2) (p 0.67) respectively. Table 4 shows the changes in physical component score and mental component score in all participants and subgroups.

**Table 4:** Changes in Physical Component Scores (PCS) and Mental Component Scores (MCS) from preoperatively to two year after Roux-en-Y Gastric bypass (RYGB)

Group (number of participants)	n	Δ PCS	95% CI	*p*-value	Δ MCS	95% CI	*p*-value
All participants	153	7.1	5.4 to 8.8	**<0.001**	−1,25	−2.9 to 0.44	0.15
Subgroups based on IBS at preoperative visit:							
With IBS	10	0.54	−8.6 to 9.7	0.90	−2.6	−8.5 to 3.2	0.33
Without IBS	143	7.6	5.8 to 9.3	**<0.001**	−1.2	−2.9 to 0.62	0.20
Subgroups based on IBS-like symptoms 2 years after RYGB:							
Never IBS or IBS-like symptoms	113	7.68	5.8 to 9.6	**<0.001**	0.29	−1.5 to 2.1	0.75
New onset IBS-like symptoms	30	7.15	2.8 to 11.5	**0.002**	−6.59	−11.3 to − 1.9	**0.007**
IBS at preoperative and IBS-like symptoms 2 year follow-up visit	6	−3.3	−14.1 to 7.5	0.47	−4.9	−14.8 to 5.0	0.26

All participants, and subgroups based on status of irritable bowel syndrome (IBS) are presented. Significant *p*-values are marked in bold

The SF-36 questionnaires were scored using the QualityMetric Health Outcomes™ Scoring Software 4.5 giving the presented Physical Component Scores (PCS) and Mental Component Scores (MCS)

The presented delta values: SF-36 value at the 2 year follow-up visit - SF-36 value at the preoperative visit. Positive delta values indicate improved score after RYGB and negative values indicate worsened score after RYGB. The results are given as mean values with 95% confidence intervals (CI). Data were analyzed with paired t-tests

New onset IBS-like symptoms: no IBS before RYGB, but IBS-like symptoms 2 years after RYGB. Never IBS or IBS-like symptoms: No IBS before or IBS-like symptoms 2 years after RYGB

Missing data: All participants; *n* = 80. Subgroups based on IBS at preoperative visit: with IBS; *n* = 17, without IBS; *n* = 63. Subgroups based on IBS-like symptoms 2 years after RYGB: never IBS or IBS-like symptoms; *n* = 48, new onset IBS-like symptoms; *n* = 15, IBS at preoperative and IBS-like symptoms 2 year follow-up visit; *n* = 10

## Discussion

The prevalence of IBS-like symptoms more than doubled 2 years after RYGB. IBS-like symptoms thus appears to be a relevant contributing factor for chronic abdominal pain and symptoms after RYGB. It has been hypothesized that low-grade inflammation could be an important cause of IBS in patients with morbid obesity [19]. In our study, the prevalence of IBS-like symptoms increased in a period where obesity-related inflammation decreased for the majority of participants, weakening this hypothesis.

Participants with preoperative fibromyalgia had four times higher odds of IBS-like symptoms after RYGB than participants without fibromyalgia. Fibromyalgia and IBS may share similar pain processing dysfunctions, such as reduced pain inhibition and aberrant autonomic nervous system responses [20–22]. The considerable weight reduction and the associated reduction of various obesity related symptoms after RYGB may impact the weighting of different bodily sensations (Table 1), and thus in combination with altered gastrointestinal physiology may affect the prevalence of perceived IBS-like symptoms.

Unexpectedly, high levels of vitamin B_1_ before RYGB was an independent preoperative predictor of IBS-like symptoms 2 year post surgery. This finding may be a proxy indicating a specific diet used by these patients or it may be a statistical type 1 bias. The finding should be further explored in future studies.

Participants with lower LDL at the preoperative visit had higher probability of IBS-like symptoms at the 2 year follow-up visit. Previous reports note an association between *higher* LDL and IBS both in subjects with and without obesity [23, 24]. RYGB induces structural changes in gastrointestinal tract, affects the regulation of several gut hormones including those involved in hunger and satiety, and has been shown to affect the responses of the cerebral frontoparietal control network involved in food preference regulation and non-food cues [25–27]. The net effect is decreased intake of food, enhanced satiety responses and a shift in food preferences from high-energy to low-energy. The pathophysiology of IBS before RYGB could thus differ from that of IBS-like symptoms post RYGB. In subjects with morbid obesity, before surgery, IBS may be more related to the diet or the lipid metabolism [24], while after RYGB IBS-like symptoms could be more related to disturbances in pain perception and altered gastrointestinal physiology.

Both constipation and diarrhea symptoms increased after RYGB, with a comparable mean change in participants with and without IBS at the preoperative visit. Previous studies have shown diverging dynamics of these symptoms after RYGB [12, 28, 29]. However, although statistically significant, the absolute changes in mean diarrhea and constipation symptom score are unlikely to be of high clinically importance (< 15% increase in symptom score), with exception of the subgroup of participants with new onset of IBS-like symptoms after RYGB who experienced a mean 33% increase for both diarrhea and constipation symptom scores.

The observed improvements in the physical functioning aspects of HRQoL after RYGB have previously been described [9, 12]. However, we are the first to describe that the improved physical functioning is irrespective of the presence of IBS-like symptoms after RYGB. Participants with and without IBS at the preoperative visit had comparable HRQoL. This was surprising, but may be due to the limited number of participants with IBS and HRQoL data available before surgery. After RYGB participants with IBS-like symptoms had significantly lower HRQoL than participants without such symptoms, in line with previous reports [30].

Of the 206 participants without IBS preoperatively 45 (22%) had new onset IBS-like symptoms 2 years after surgery. This group had a larger increase in diarrhea and constipation symptom scores and a decrease in the mental component score of SF-36 after RYGB. These findings supports an association between IBS-like symptoms after RYGB and worsened psychosocial functioning, and highlight the impact IBS-like symptoms may have on patients’ lives after RYGB [31]. The high rate of IBS-like symptoms after surgery and the reduced HRQoL associated with such symptoms should be part of the preoperative information to patients eligible for bariatric surgery and a clinical focus during follow-up, including guidance in how to reduce symptoms.

Participants with IBS before surgery had seven times higher odds for IBS-like symptoms 2 years after RYGB than those without IBS prior to surgery. However, in participants with IBS preoperatively we observed unchanged HRQoL after RYGB. And it is worth to point out that of the 27 participants with IBS preoperatively 11 (41%) no longer had IBS-like symptoms 2 years after RYGB. Therefore, IBS prior to RYGB should not be considered as an absolute contraindication for surgery.

The large numbered cohort, the high rate of follow up, and the prospective design are important strengths of our study compared to earlier reports. The use of a validated translation of the Rome III and the SF-36 questionnaires and the comprehensive evaluation of all participants are other strengths.

This study contains several major limitations. The study did not differentiate between the different functional abdominal pain syndromes. Participants fulfilling the Rome III criteria for IBS were not systematically examined for underlying pathophysiology of IBS-like symptoms. To what extent the altered physiology or other aspects of the surgical procedure itself contributed to the increase in bowel symptoms could not be answered by this study. In particular, evaluation of small intestinal bacterial overgrowth would be relevant in the evaluation of symptoms. Reporting on the use of medication at follow-up may have added information relevant for interpretation of our findings, particularly medications affecting gastrointestinal function. A control group consisting of patients undergoing other abdominal surgery could add information relevant to the interpretation of our findings.

The large number of variables imposes a risk of type 1 statistical errors. Correction for multiple testing was not performed, but all analyzed variables are presented transparently. Only participants from OUS were evaluated with SF-36 preoperatively. Gastrointestinal symptoms were evaluated with two subtypes of GSRS, thus limiting the study to only report diarrhea and constipation symptom scores for which questions in the two questionnaires were identical. A more precise diagnosis of psychiatric disorders could have strengthened the study.

Blood tests were analyzed at two local laboratories. However for paired sample tests a difference between the laboratories would not affect the results, as all patients were seen at the same center at the preoperative and the 2 year follow-up visits.

## Conclusions

The prevalence of IBS-like symptoms more than doubled and was associated with reduced HRQoL after RYGB. Preoperative IBS and fibromyalgia were strong predictors of such symptoms 2 years after RYGB, yet most of the patients with IBS-like symptoms after surgery had new onset of symptoms.

## Data Availability

The data that support the findings of this study have been used according to the approval from Regional Committee for Medical and Health Research Ethics South East Norway and are not publicly available. Data can however be made available from the corresponding authors upon reasonable request and after permission of Regional Committee for Medical and Health Research Ethics South East Norway has been granted.

## Abbreviations

BMI: Body mass index
CRP: C-reactive protein
GSRS: Gastrointestinal Symptoms Rating Scale
HbA_1c_: Glycosylated hemoglobin
HDL: High-density lipoprotein
HRQoL: Health related quality of life
IBS: Irritable bowel syndrome
IHT-G: Innlandet Hospital Trust Gjøvik
LDL: Low-density lipoprotein (LDL)
OUH: Oslo University Hospital
RYGB: Roux-en-Y gastric bypass
SF-36: 36-Item Short Form Health Survey (SF-36)
T2D: Type 2 diabetes
TSH: Thyroid stimulating hormone

## References

[1] Sperber Ami D, Dumitrascu Dan, Fukudo Shin, Gerson Charles, Ghoshal Uday C, Gwee Kok Ann, Hungin A Pali S, Kang Jin-Yong, Minhu Chen, Schmulson Max, Bolotin Arkady, Friger Michael, Freud Tamar, Whitehead William (2016). The global prevalence of IBS in adults remains elusive due to the heterogeneity of studies: a Rome Foundation working team literature review. Gut.

[2] Fysekidis Marinos, Bouchoucha Michel, Bihan Hélène, Reach Gérard, Benamouzig Robert, Catheline Jean-Marc (2011). Prevalence and Co-occurrence of Upper and Lower Functional Gastrointestinal Symptoms in Patients Eligible for Bariatric Surgery. Obesity Surgery.

[3] Bouchoucha Michel, Fysekidis Marinos, Julia Chantal, Airinei Gheorghe, Catheline Jean-Marc, Reach Gérard, Benamouzig Robert (2015). Functional Gastrointestinal Disorders in Obese Patients. The Importance of the Enrollment Source. Obesity Surgery.

[4] Ford Alexander C., Lacy Brian E., Talley Nicholas J. (2017). Irritable Bowel Syndrome. New England Journal of Medicine.

[5] Longstreth George F., Thompson W. Grant, Chey William D., Houghton Lesley A., Mearin Fermin, Spiller Robin C. (2006). Functional Bowel Disorders. Gastroenterology.

[6] Angrisani L., Santonicola A., Iovino P., Vitiello A., Zundel N., Buchwald H., Scopinaro N. (2017). Bariatric Surgery and Endoluminal Procedures: IFSO Worldwide Survey 2014. Obesity Surgery.

[7] Courcoulas Anita P., King Wendy C., Belle Steven H., Berk Paul, Flum David R., Garcia Luis, Gourash William, Horlick Mary, Mitchell James E., Pomp Alfons, Pories Walter J., Purnell Jonathan Q., Singh Ashima, Spaniolas Konstantinos, Thirlby Richard, Wolfe Bruce M., Yanovski Susan Z. (2018). Seven-Year Weight Trajectories and Health Outcomes in the Longitudinal Assessment of Bariatric Surgery (LABS) Study. JAMA Surgery.

[8] Suter Michel, Donadini Andrea, Romy Sébastien, Demartines Nicolas, Giusti Vittorio (2011). Laparoscopic Roux-En-Y Gastric Bypass. Annals of Surgery.

[9] Risstad Hilde, Søvik Torgeir T., Hewitt Stephen, Kristinsson Jon A., Fagerland Morten W., Bernklev Tomm, Mala Tom (2015). Changes in Health-Related Quality of Life After Gastric Bypass in Patients With and Without Obesity-Related Disease. Obesity Surgery.

[10] Høgestøl Ingvild K., Chahal-Kummen Monica, Eribe Inger, Brunborg Cathrine, Stubhaug Audun, Hewitt Stephen, Kristinsson Jon, Mala Tom (2016). Chronic Abdominal Pain and Symptoms 5 Years After Gastric Bypass for Morbid Obesity. Obesity Surgery.

[11] Gribsholt Sigrid Bjerge, Pedersen Ane Mathilde, Svensson Elisabeth, Thomsen Reimar Wernich, Richelsen Bjørn (2016). Prevalence of Self-reported Symptoms After Gastric Bypass Surgery for Obesity. JAMA Surgery.

[12] Chahal-Kummen MIB-H, Eribe I, Klungsøyr O, Kristinsson J, Mala T (2019). Abdominal pain and symptoms before and two years after roux-en-Y gastric bypass. BJS Open.

[13] Pernar Luise I. M., Lockridge Ryan, McCormack Colleen, Chen Judy, Shikora Scott A., Spector David, Tavakkoli Ali, Vernon Ashley H., Robinson Malcolm K. (2016). An Effort to Develop an Algorithm to Target Abdominal CT Scans for Patients After Gastric Bypass. Obesity Surgery.

[14] Blom-Høgestøl Ingvild K., Stubhaug Audun, Kristinsson Jon A, Mala Tom (2018). Diagnosis and treatment of chronic abdominal pain 5 years after Roux-en-Y gastric bypass. Surgery for Obesity and Related Diseases.

[15] Pierik Annouk S., Coblijn Usha K., de Raaff Christel A.L., van Veen R.N., van Tets Willem F., van Wagensveld Bart A. (2017). Unexplained abdominal pain in morbidly obese patients after bariatric surgery. Surgery for Obesity and Related Diseases.

[16] Schauer PR, Ikramuddin S, Hamad G, Eid GM, Mattar S, Cottam D, et al. Laparoscopic gastric bypass surgery: current technique. Journal of laparoendoscopic & advanced surgical techniques Part A 2003;13(4):229–239. PubMed PMID: 14561251. Epub 2003/10/17. eng. 10.1089/109264203322333557 14561251

[17] Svedlund J, Sjodin I, Dotevall G. GSRS--a clinical rating scale for gastrointestinal symptoms in patients with irritable bowel syndrome and peptic ulcer disease. Dig Dis Sci 1988;33(2):129–134. PubMed PMID: 3123181. Epub 1988/02/01. eng. 10.1007/BF01535722 3123181

[18] Karlsen Tor-Ivar, Tveitå Einar K., Natvig Gerd K., Tonstad Serena, Hjelmesæth Jøran (2011). Validity of the SF-36 in Patients with Morbid Obesity. Obesity Facts.

[19] Pickett-Blakely O. Obesity and irritable bowel syndrome: a comprehensive review. Gastroenterol Hepatol 2014;10(7):411–416. PubMed PMID: 25904828. Pubmed Central PMCID: PMC4302488. Epub 2015/04/24. eng. PMC4302488 25904828

[20] Kurland Jayde E., Coyle Walter J., Winkler Anne, Zable Elizabeth (2006). Prevalence of Irritable Bowel Syndrome and Depression in Fibromyalgia. Digestive Diseases and Sciences.

[21] VEALE D., KAVANAGH G., FIELDING J. F., FITZGERALD O. (1991). PRIMARY FIBROMYALGIA AND THE IRRITABLE BOWEL SYNDROME: DIFFERENT EXPRESSIONS OF A COMMON PATHOGENETIC PROCESS. Rheumatology.

[22] Chalaye Philippe, Goffaux Philippe, Bourgault Patricia, Lafrenaye Sylvie, Devroede Ghislain, Watier Alain, Marchand Serge (2012). Comparing Pain Modulation and Autonomic Responses in Fibromyalgia and Irritable Bowel Syndrome Patients. The Clinical Journal oF Pain.

[23] Gulcan Erim, Taser Figen, Toker Aysun, Korkmaz Ugur, Alcelik Aytekin (2009). Increased Frequency of Prediabetes in Patients With Irritable Bowel Syndrome. The American Journal of the Medical Sciences.

[24] Aasbrenn M, Hogestol I, Eribe I, Kristinsson J, Lydersen S, Mala T, et al. Prevalence and predictors of irritable bowel syndrome in patients with morbid obesity: a cross-sectional study. BMC obesity 2017;4:22. PubMed PMID: 28680646. Pubmed Central PMCID: PMC5490229. Epub 2017/07/07. eng. 10.1186/s40608-017-0159-z PMC5490229 28680646

[25] Goldstone Anthony P., Miras Alexander D., Scholtz Samantha, Jackson Sabrina, Neff Karl J., Pénicaud Luc, Geoghegan Justin, Chhina Navpreet, Durighel Giuliana, Bell Jimmy D., Meillon Sophie, le Roux Carel W. (2016). Link Between Increased Satiety Gut Hormones and Reduced Food Reward After Gastric Bypass Surgery for Obesity. The Journal of Clinical Endocrinology & Metabolism.

[26] Zoon Harriët F.A., de Bruijn Suzanne E.M., Smeets Paul A.M., de Graaf Cees, Janssen Ignace M.C., Schijns Wendy, Aarts Edo O., Jager Gerry, Boesveldt Sanne (2018). Altered neural responsivity to food cues in relation to food preferences, but not appetite-related hormone concentrations after RYGB-surgery. Behavioural Brain Research.

[27] Kvehaugen AS, Farup PG. Changes in gastrointestinal symptoms and food tolerance 6 months following weight loss surgery: associations with dietary changes, weight loss and the surgical procedure. BMC obesity 2018;5:29. PubMed PMID: 30524734. Pubmed Central PMCID: PMC6276242. Epub 2018/12/14. eng. 10.1186/s40608-018-0206-4 PMC6276242 30524734

[28] Søvik Torgeir T., Karlsson Jan, Aasheim Erlend T., Fagerland Morten W., Björkman Sofia, Engström My, Kristinsson Jon, Olbers Torsten, Mala Tom (2013). Gastrointestinal function and eating behavior after gastric bypass and duodenal switch. Surgery for Obesity and Related Diseases.

[29] Clements Ronald H., Gonzalez Quintin H., Foster Allen, Richards William O., McDowell James, Bondora Anthony, Laws Henry L. (2003). Gastrointestinal Symptoms are More Intense in Morbidly Obese Patients and are Improved with Laparoscopic Roux-en-Y Gastric Bypass. Obesity Surgery.

[30] Nellesen Dave, Yee Kimberly, Chawla Anita, Lewis Barbara Edelman, Carson Robyn T. (2013). A Systematic Review of the Economic and Humanistic Burden of Illness in Irritable Bowel Syndrome and Chronic Constipation. Journal of Managed Care Pharmacy.

[31] Przekop P, Haviland MG, Zhao Y, Oda K, Morton KR, Fraser GE. Self-reported physical health, mental health, and comorbid diseases among women with irritable bowel syndrome, fibromyalgia, or both compared with healthy control respondents. The Journal of the American Osteopathic Association 2012;112(11):726–735. PubMed PMID: 23139343. Pubmed Central PMCID: PMC3542981. Epub 2012/11/10. eng. PMC3542981 23139343

